# The role of nurse‐led telehealth interventions in bridging healthcare gaps and expanding access

**DOI:** 10.1002/nop2.2092

**Published:** 2024-01-11

**Authors:** Lemma N. Bulto

**Affiliations:** ^1^ Caring Futures Institute, College of Nursing and Health Sciences Flinders University Adelaide South Australia Australia

**Keywords:** health sercice, nurse, nurse‐led, telehealth

Nurse‐led telehealth interventions involve the use of technology to enable nurses to deliver healthcare services remotely using technology such as phone, video conferencing and remote monitoring devices, bridging the gap between patients and providers (Barton et al., 2023; Joo, 2022). As the global healthcare landscape evolves, nurse‐led telehealth interventions have gained attention for their ability to provide timely, patient‐centred care while addressing issues of accessibility and convenience (Joo, 2022). The rapid advancement of telehealth technologies has transformed the landscape of healthcare delivery, offering innovative solutions to overcome barriers related to geographical distance, limited access, and time constraints (Macduff et al., 2001; MacKenzie et al., 2010; Mizukawa et al., 2019). Among these novel approaches, nurse‐led telehealth interventions have emerged as a promising avenue to improve patient outcomes, enhance patient engagement, and optimize resource utilization (Joo, 2022; Kappes et al., 2023). This editorial explores the significance of nurse‐led telehealth interventions, their impact on healthcare delivery, and the potential challenges and benefits associated with their implementation.

## 1 ADVANTAGES OF NURSE‐LED TELEHEALTH INTERVENTIONS

Nurse‐led telehealth interventions surpass geographical barriers, allowing patients, especially those in remote or underserved areas, to access expert care without the need for extensive travel (Barton et al., 2023; Koh et al., 2016; Lee et al., 2022). For example, a recent systematic review highlights the latest evidence on nurse‐led telehealth interventions used to effectively reduce blood pressure and modify health behaviours among patients (Bulto et al., 2023). Telehealth interventions also empower nurses to monitor patients with chronic conditions regularly, providing timely interventions and reducing hospitalizations. In addition, nurses can use telehealth to educate patients about their conditions, medications, and self‐care, fostering a sense of empowerment and ownership over their health (Koh et al., 2016; Kwok et al., 2022). Nurse‐led telehealth has also contributed to cost savings for both healthcare institutions and patients by minimizing unnecessary hospital visits and readmissions. Furthermore, telehealth allows nurses to detect potential health issues early, enabling timely interventions and promoting preventive care strategies.

## 2 IMPACT ON HEALTHCARE DELIVERY

Nurse‐led telehealth interventions can be used to prioritize patient needs, offering tailored care plans that align with individual preferences and circumstances. Patients can maintain a consistent relationship with their designated nurse, leading to better communication and care coordination. By reaching patients who face barriers to traditional healthcare access such as transportation, financial and time constraints and lack of access, nurse‐led telehealth interventions contribute to more equitable healthcare delivery (Tietjen & Breitenstein, 2017). Telehealth helps optimize healthcare resources by redirecting in‐person appointments to more critical cases and streamlining routine care. In addition, nurse‐led telehealth interventions generate valuable patient data that can inform evidence‐based practice and policy decisions, by enabling recording of patient's data such as clinical data and other data related to health behaviour.

## 3 CHALLENGES AND CONSIDERATIONS

Ensuring patient and nurse proficiency with telehealth platforms and devices is essential for effective implementation. Safeguarding patient information and complying with data protection regulations are critical to maintaining trust in telehealth services. Disparities in technology access and digital literacy may exclude certain populations from the benefits of nurse‐led telehealth interventions (Lee et al., 2022). Building rapport and trust remotely can be challenging, emphasizing the need for effective communication strategies. Nurse‐led telehealth interventions must adhere to local and national regulations, which can vary and impact service delivery. Nurse scope of practice in nurse‐led interventions covers healthcare activities initiated and implemented by nurses, often without direct physician overseeing, to promote patient well‐being and optimize patient outcomes. Emerging nurse‐led models of care, coordinated primarily by registered nurses, advanced practice nurses, and/or nurse practitioners, are facilitating the implementation of nurse‐led interventions including nurse‐led telehealth, that involve patients and their families/carers in managing their health conditions (Bulter et al., 2020).

## 4 POLICY AND PRACTICAL IMPLICATIONS

Nurse‐led telehealth interventions present a transformative avenue in healthcare delivery, influencing both policy formulation and practical implementation. Policymakers must navigate the complex terrain of licensure, reimbursement, and data security while fostering an environment conducive to collaborative practice. Concurrently, nursing professionals must embrace evolving roles and develop requisite skills to effectively leverage technology in patient care, while healthcare institutions should prioritize the technological infrastructure and quality assurance mechanisms necessary for sustained success. Through careful consideration of these policy and practical dimensions, nurse‐led telehealth interventions can be used to enhance patient outcomes and reshape the delivery of contemporary healthcare.

In conclusion, nurse‐led telehealth interventions represent an innovative approach to healthcare delivery that leverages technology to bridge gaps in access, improve patient outcomes, and optimize resource utilization. As the healthcare landscape continues to evolve, the integration of nurse‐led telehealth interventions holds the potential to enhance patient care, empower individuals, and foster a more equitable and patient‐centred healthcare system. Addressing challenges through collaborative efforts among healthcare professionals, policymakers, and technology providers will be essential for realizing the full benefits of nurse‐led telehealth interventions.

## AUTHOR CONTRIBUTIONS

LNB conceptualized and wrote the paper.

## CONFLICT OF INTEREST STATEMENT

No conflict of interest.

## FUNDING INFORMATION

No funding received.

## Data Availability

Data sharing not applicable to this article as no datasets were generated or analysed during the current study.

## References

[nop22092-bib-0001] Barton, A. J. , Amura, C. R. , Willems, E. L. , Medina, R. , Centi, S. , Hernandez, T. , Reed, S. M. , & Cook, P. F. (2023). Patient and provider perceptions of COVID‐19‐driven telehealth use from nurse‐led care models in rural, frontier, and urban Colorado communities. Journal of Patient Experience, 10, 23743735231151546. 10.1177/23743735231151546 36741820 PMC9893383

[nop22092-bib-0002] Bulter, M. , Schultz, J. , & Drennan, J. (2020). Substitution of nurses for physicians in the hospital setting for patient, process of care, and economic outcomes. Cochrane Database of Systematic Reviews, 5, 13616. 10.1002/14651858.CD013616 PMC12893813 41672426

[nop22092-bib-0003] Bulto, L. N. , Roseleur, J. , Noonan, S. , Pinero de Plaza, M. A. , Champion, S. , Dafny, H. A. , Pearson, V. , Nesbitt, K. , Gebremichael, L. G. , Beleigoli, A. , Schultz, T. , Hines, S. , Clark, R. A. , & Hendriks, J. M. (2023). Effectiveness of nurse‐led interventions versus usual care to manage hypertension and lifestyle behaviour: A systematic review and meta‐analysis. European Journal of Cardiovascular Nursing. 10.1093/eurjcn/zvad040 37130339

[nop22092-bib-0004] Joo, J. Y. (2022). Nurse‐led telehealth interventions during COVID‐19: A scoping review. Computers, Informatics, Nursing, 40(12), 804–813. 10.1097/cin.0000000000000962 PMC9762614 36067472

[nop22092-bib-0005] Kappes, M. , Espinoza, P. , Jara, V. , & Hall, A. (2023). Nurse‐led telehealth intervention effectiveness on reducing hypertension: A systematic review. BMC Nursing, 22(1), 19. 10.1186/s12912-022-01170-z 36650463 PMC9843665

[nop22092-bib-0006] Koh, K. W. , Wang, W. , Richards, A. M. , Chan, M. Y. , & Cheng, K. K. (2016). Effectiveness of advanced practice nurse‐led telehealth on readmissions and health‐related outcomes among patients with post‐acute myocardial infarction: ALTRA study protocol. Journal of Advanced Nursing, 72(6), 1357–1367. 10.1111/jan.12933 26915719

[nop22092-bib-0007] Kwok, C. , Degen, C. , Moradi, N. , & Stacey, D. (2022). Nurse‐led telehealth interventions for symptom management in patients with cancer receiving systemic or radiation therapy: A systematic review and meta‐analysis. Support Care Cancer, 30(9), 7119–7132. 10.1007/s00520-022-07052-z 35420331 PMC9008678

[nop22092-bib-0008] Lee, A. Y. L. , Wong, A. K. C. , Hung, T. T. M. , Yan, J. , & Yang, S. (2022). Nurse‐led telehealth intervention for rehabilitation (telerehabilitation) among community‐dwelling patients with chronic diseases: Systematic review and meta‐analysis. Journal of Medical Internet Research, 24(11), e40364. 10.2196/40364 36322107 PMC9669889

[nop22092-bib-0009] Macduff, C. , West, B. , & Harvey, S. (2001). Telemedicine in rural care. Part 2: Assessing the wider issues. Nursing Standard, 15(33), 33–37. 10.7748/ns2001.05.15.33.33.c3020 12216265

[nop22092-bib-0010] MacKenzie, E. , Smith, A. , Angus, N. , Menzies, S. , Brulisauer, F. , & Leslie, S. J. (2010). Mixed‐method exploratory study of general practitioner and nurse perceptions of a new community based nurse‐led heart failure service. Rural and Remote Health, 10(4), 1510. 21028933

[nop22092-bib-0011] Mizukawa, M. , Moriyama, M. , Yamamoto, H. , Rahman, M. M. , Naka, M. , Kitagawa, T. , Kobayashi, S. , Oda, N. , Yasunobu, Y. , Tomiyama, M. , Morishima, N. , Matsuda, K. , & Kihara, Y. (2019). Nurse‐led collaborative management using telemonitoring improves quality of life and prevention of rehospitalization in patients with heart failure. International Heart Journal, 60(6), 1293–1302. 10.1536/ihj.19-313 31735786

[nop22092-bib-0012] Tietjen, K. M. , & Breitenstein, S. (2017). A nurse‐led telehealth program to improve emotional health in individuals with multiple sclerosis. Journal of Psychosocial Nursing and Mental Health Services, 55(3), 31–37. 10.3928/02793695-20170301-04 28287673

